# Cerium-Doped CuFe-Layered Catalyst for the Enhanced Oxidation of *o*-Xylene and *N*,*N*-Dimethylacetamide: Insights into the Effects of Temperature and Space Velocity

**DOI:** 10.1021/acsomega.3c05175

**Published:** 2023-11-20

**Authors:** Zehra Betul Ocal, Ramazan Keyikoglu, Ahmet Karagunduz, Yeojoon Yoon, Alireza Khataee

**Affiliations:** † Department of Environmental Engineering, Gebze Technical University, 41400 Gebze, Turkey; ‡ Department of Environmental Engineering, Sinop University, 57000 Sinop, Turkey; § Department of Environmental Engineering, Bursa Technical University, 16310 Bursa, Turkey; ∥ Department of Environmental and Energy Engineering, Yonsei University, 26493Wonju, Republic of Korea; ⊥ Research Laboratory of Advanced Water and Wastewater Treatment Processes, Department of Applied Chemistry, Faculty of Chemistry, University of Tabriz, 51666−16471 Tabriz, Iran

## Abstract

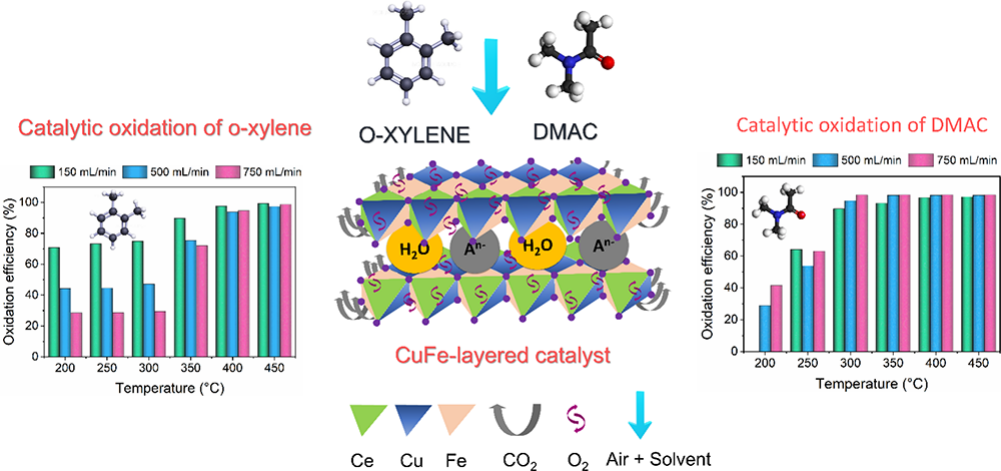

Volatile organic compounds (VOCs) are among the most potential pollutant groups that cause air quality degradation because of their toxic effects on human health. Although catalytic oxidation is an effective method for VOC removal, further studies are required to develop more efficient and affordable catalysts. In this study, cerium (Ce) was doped into a CuFe-layered material (Ce–CuFe) to improve the catalytic oxidation efficiencies of *N*,*N*-dimethylacetamide (DMAC) and *o*-xylene. The synthesized catalyst was characterized using X-ray diffraction (XRD), scanning electron microscopy (SEM), high-resolution transmission electron microscopy (HR-TEM), energy-dispersive X-ray spectroscopy (EDS), Fourier-transform infrared spectroscopy (FTIR), and X-ray photoelectron spectroscopy (XPS) analysis. XRD analysis confirmed the successful doping of Ce atoms into the CuFe-layered structure, while in the SEM and TEM images the catalyst appeared as uniformly distributed two-dimensional plate-like particles. The catalytic oxidation performance of the Ce–CuFe was investigated at six temperatures between 200 and 450 °C and three space velocities in the range of 31000–155000 mLh^–1^g^–1^ for the oxidation of DMAC and *o*-xylene, which functioned as polar and nonpolar solvents, respectively. At 200 °C, the Ce–CuFe catalyst performed 50% greater when oxidizing *o*-xylene while exhibiting a DMAC oxidation efficiency that was 42% greater than that achieved using undoped CuFe. The Ce–CuFe could remove DMAC and *o*-xylene with an efficiency higher than 95% at 450 °C. Furthermore, Ce-doped CuFe exhibited high resistance against moisture and outstanding reusability performance with only a 5.6% efficiency loss after nine reuse cycles. Overall, the incorporation of Ce into a CuFe-layered material is a promising strategy for the oxidation of various VOCs.

## 1 Introduction

Volatile organic compounds (VOCs) are chemical compounds containing at least one carbon and hydrogen in their structures that evaporate rapidly in direct contact with air at room temperature. VOCs are hazardous indoor pollutants that are caused by fossil fuels, industrial processes, biofuel combustion, biomass burning, and waste management.^1^ Various methods are available for recovering and removing VOCs from the air, such as thermal oxidation, catalytic oxidation, adsorption, absorption, biofiltration, membrane separation, and plasma technology.^2^ Although thermal oxidation is very effective for VOC removal, its high cost is a major limiting factor. Meanwhile, the biofiltration method, in which microorganisms degrade organic materials, requires additional nutrients and results in sludge production.^3^ Although adsorption and membrane processes are very effective in removing VOCs from gas streams, they only transfer pollutants from one phase to another because of their nondestructive nature.

Catalytic oxidation is one of the most commonly used methods to remove VOCs from gas streams.^4^ Noble metallic catalysts provide excellent oxidation efficiencies at low temperatures. Studies performed with Pt-doped zeolite and Pd-doped Al_2_O_3_ achieved 100% *o*-xylene oxidation at 220 °C and temperatures below 140 °C, respectively.^5,6^ However, studies have focused on non-noble metal-based catalysts that can offer the same efficiency at a reasonable cost.^7^ In particular, catalysts containing transition metals on porous supports exhibited promising activities owing to their better dispersibility and longer retention of VOCs on their surfaces.^8^ Cerium (Ce) possesses high redox properties^9^ accompanied by a high oxygen storage capacity^10^ that makes it a potential active ingredient for VOC oxidation. Ce-based catalysts have shown effectiveness in various catalytic processes, such as hydrothermal NO_*x*_ aging,^11^ anthraquinone hydrogenation,^12^ ethane dehydrogenation,^13^ depolymerization,^14^ methanol steam reforming,^15^ catalytic ozonation,^16^ and hydrothermal liquefaction of granular bacteria in the production of high-quality bio-oil.^17^

Layered materials are solids with highly anisotropic bonding, where two-dimensional sheets are strongly bonded internally and weakly bonded to neighboring layers.^18^ They are made up of nanometer-thick inorganic crystalline sheets that are only loosely connected by electrostatic, van der Waals, or hydrogen-bonding interactions.^19^ The structural arrangement of these materials enables the insertion of atomic or molecular guest species between the layers.^20^ Layered double hydroxides (LDH), a special type of layered material, are classified as anionic clay or hydrotalcite-like compounds that are composed of exchangeable interlayer anions located between the cationic metal hydroxide layers.^21^ A wide range of LDHs with unique properties can be formed by changing their compositions. Calcination of LDHs is a common approach for preparing layered metal oxides (LDOs) with homogeneous metal distributions and well-developed porosities.^22^

*N*,*N*-Dimethylacetamide (DMAC), a type of VOC, is a polar, colorless, and hygroscopic liquid industrial solvent that has been widely used in agrochemicals, pharmaceuticals, coatings, paint strippers, varnishes, and other applications.^23^ DMAC is resistant to degradation, and its high toxicity poses a risk to the environment and public health.^24^ The literature lacks studies investigating the removal of gaseous DMAC by physical and chemical processes including catalytic oxidation. The trickle-bed air biofilter (TBAB) process achieved 90% DMAC removal efficiency.^25^ Xylene has three isomers (*p*-xylene, *o*-xylene, and *m*-xylene), each containing two methyl groups positioned at distinct locations on a benzene ring. These colorless isomers emit a pleasant aromatic odor^26^ and can have various effects on the human body, primarily impacting the digestive, nervous, and respiratory systems.^27^ Due to their nonpolar properties, xylene isomers find extensive utility as solvents in industries such as printing, rubber production, paint manufacturing, and leather processing. Specifically, *o*-xylene is employed for applications such as plastifiers, polyesters, medicines, and agriculture.^28^ Commonly used processes for *o*-xylene removal are adsorption^29^ biodegradation,^30^ and oxidation.^31^

A few studies have used LDH-based catalysts to investigate the oxidation of different types of VOCs. To the best of our knowledge, the catalytic oxidation of polar VOCs, such as DMAC, using LDH or LDH-derived catalysts has not been investigated so far. Therefore, a further understanding of the effectiveness of LDH-based catalysts in the oxidation of VOCs with diverse chemical properties must be developed. The main aim of this study was to develop a highly efficient catalyst for the catalytic oxidation of VOCs with different polarities and properties. For this purpose, Ce was incorporated into a CuFe-layered catalyst (Ce–CuFe) using a coprecipitation process. The morphology, composition, and structural characteristics of the Ce–CuFe catalyst were evaluated by scanning electron microscopy (SEM), transmission electron microscopy (TEM), X-ray photoelectron spectroscopy (XPS), X-ray diffraction (XRD), and Fourier-transform infrared (FTIR) analyses. The performances of the pristine and Ce-doped CuFe catalysts were assessed for the catalytic oxidation of DMAC and *o*-xylene as polar and nonpolar VOCs, respectively. The oxidation performance of the catalysts was investigated at different temperatures and space velocities for the individual removal of DMAC and *o*-xylene. Finally, the durability of the Ce–CuFe catalyst was determined by multiple reuse experiments and poststructural characterization studies.

## 2 Materials and Methods

### 2.1 Materials

Iron (III) nitrate nonahydrate (Fe(NO_3_)_3_.9H_2_O, ≥ 98%), copper (II) nitrate trihydrate (Cu(NO_3_)_2_.3H_2_O, ≥ 99%), cerium (III) nitrate hexahydrate (Ce(NO_3_)_3_.6H_2_O), and sodium hydroxide (NaOH, ≥ 97.0%) were purchased from Sigma-Aldrich Chemie GmbH, Germany. DMAC (<99%) as a polar solvent and *o*-xylene (<99%) as a nonpolar solvent were purchased from Merck KGaA, Germany.

### 2.2 Synthesis of CuFe and Ce-Doped CuFe Catalysts

The CuFe catalyst was prepared using a coprecipitation technique.^21^ For this purpose, 3 mmol of Cu(NO_3_)_2_·3H_2_O and 1 mmol of Fe(NO_3_)_3_·9H_2_O were dissolved in 40 mL of distilled water that was purged with N_2_ gas. The pH of the solution was slowly increased to 8 by using 2 M NaOH under vigorous stirring. The synthesis of the catalyst was carried out under a nitrogen atmosphere, including a 24 h aging period to prevent unwanted reactions or oxidation of the materials. The solid samples were separated from the solution using a centrifuge at 5000 rpm and washed three times, first with ethanol and then twice with Milli-Q water to remove unprecipitated species. Finally, samples were dried by heating in an oven at 50 °C before being ground into fine powders. For the synthesis of the Ce-doped CuFe catalyst, 3 mmol of Cu(NO_3_)_2_·3H_2_O, 0.5 mmol of Fe(NO_3_)_3_·9H_2_O, and 0.5 mmol of Ce(NO_3_)_3_·6H_2_O were used, and the remaining procedures were the same as those used for the synthesis of the undoped CuFe catalyst.

### 2.3 Characterization of Ce–CuFe Catalyst

The crystalline nature of the Ce–CuFe catalyst was determined by using XRD (Tongda-TD-3700, China) with Cu Kα radiation (λ = 1.5406 Å; 30 kV, 20 mA). The surface properties were studied using a scanning electron microscope (Tescan Mira3 microscope, Czech Republic) and a TEM microscope operated at 200 kV (JEOL JEM-2100 Plus, Japan). During SEM analysis, a thin layer of gold was coated onto the sample surface under vacuum conditions using a dedicated coater. The sample was imaged using the SEM detectors at 15kV of accelerating voltage. For TEM analysis, the sample initially was dispersed in an ethanol solvent and then placed onto a TEM grid, which was introduced into the TEM chamber under a high vacuum, where the samples were imaged using TEM at suitable magnifications and operational parameters. Energy-dispersive X-ray spectroscopy (EDS) was used to determine the elemental compositions of the samples (Tescan Mira3 microscope, Czech Republic). To identify the functional groups and interactions between the layers of the Ce–CuFe catalyst, an FTIR instrument using the KBr pellet technique was used (Tensor 27, Bruker, Germany). The surface chemistry of the materials was analyzed using XPS (Thermo VG K Alpha+, Thermo Fisher, USA).

### 2.4 Catalytic Oxidation Experiments

**Figure 1:**
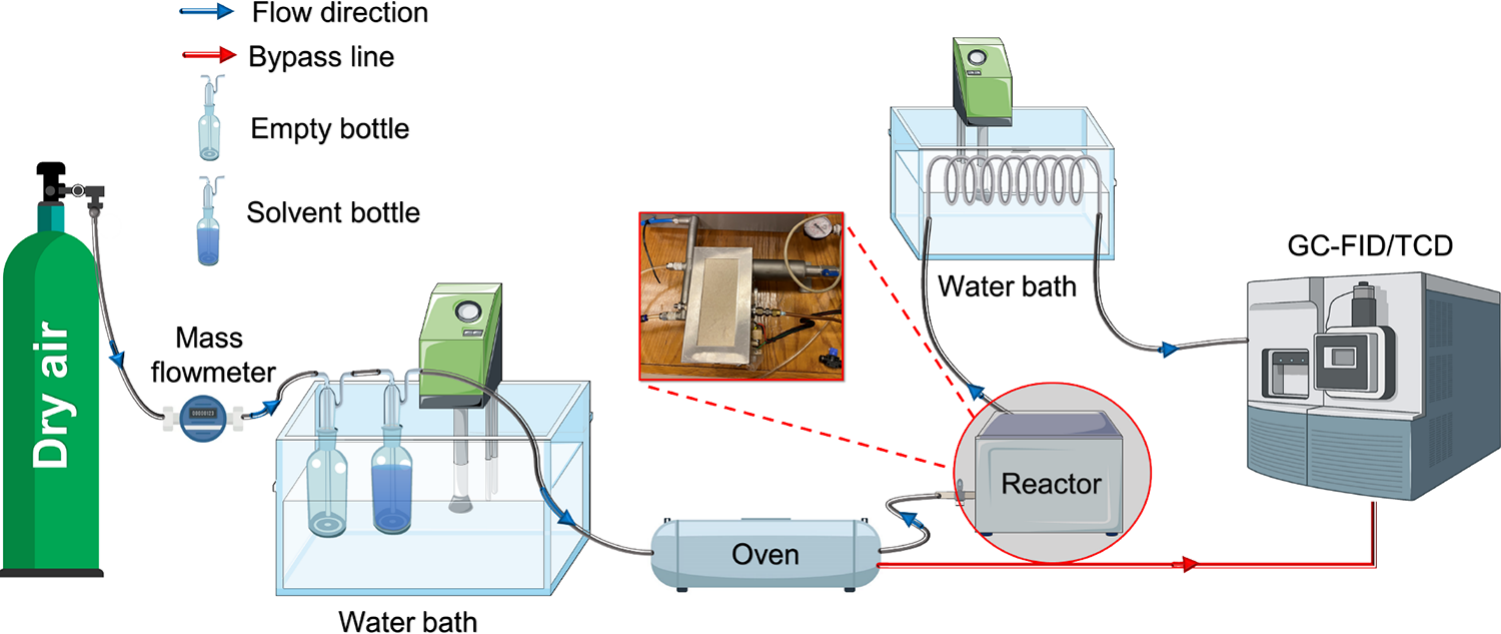
Experimental setup for catalytic oxidation of *N*,*N*-dimethylacetamide (DMAC) and *o*-xylene in the presence of Ce-doped CuFe and CuFe (the Figure was partly generated using Servier Medical Art, provided by Servier, licensed under a Creative Commons Attribution 3.0 unported license).

The experimental setup used for the study of catalytic oxidation is illustrated in Figure 1. The flow rate of dry air in the reactor was adjusted using a mass flowmeter (Bronkhorst M19204937A). The DMAC and *o*-xylene space velocities were adjusted to 31000 (150 mL/min), 103500 (500 mL/min), and 155000 mLh^–1^g^–1^ (750 mL/min). To obtain the gas phase concentration at vapor pressure, dry air was passed through a gas wash bottle containing liquid solvent (DMAC or *o*-xylene), which was placed in a constant temperature bath at 20 °C. The gas phase of the solvent and air mixture was first fed into a preheated oven. The initial concentrations of DMAC and *o*-xylene were 1740 and 6536 ppm, respectively. The preheated solvent–air mixture was fed into the reactor where the catalyst was placed between activated carbon fabric and quartz wool at both ends. The experiments were carried out in the temperature range of 200–450 °C. The gas stream exiting the reactor was cooled to 20 °C using a water bath, and a bypass line was established to determine the initial concentration of the solvent that was not in contact with the catalyst. The solvent–gas mixture was then transferred to a gas chromatograph (GC) instrument (Agilent Technologies 6890 N) with a flame ionization detector (GC–FID) and thermal conductivity detector (GC–TCD). The oven and detector temperatures were 160 and 250 °C, the flowrates of hydrogen gas and dry air were 30 and 300 mL/min, respectively, and the flow rate of nitrogen, which was used as the carrier gas, was 35 mL/min. To calculate the oxidation efficiency, eq 1 was used.
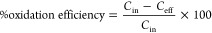
1where *C*_in_ is the initial concentration of solvent and *C*_eff_ is the effluent concentration of solvent.

## 3 Results and Discussion

### 3.1 Characterization of CuFe and Ce–CuFe catalysts

**Figure 2:**
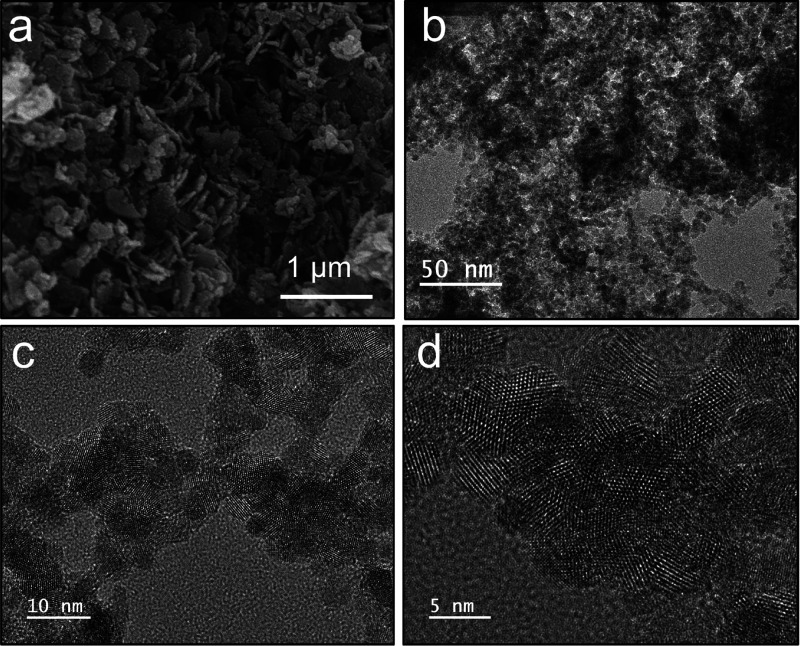
(a, b) Scanning electron microscopy (SEM) and (c, d) high-resolution transmission electron microscopy (TEM) images of Ce–CuFe.

SEM microscopy showed that the Ce–CuFe catalyst consisted of evenly distributed flakes, confirming the characteristic of layered materials (Figure 2a,b).^21^ Similarly, the formation of plate-like layered particles was observed in the TEM images (Figure 2c,d).

**Figure 3:**
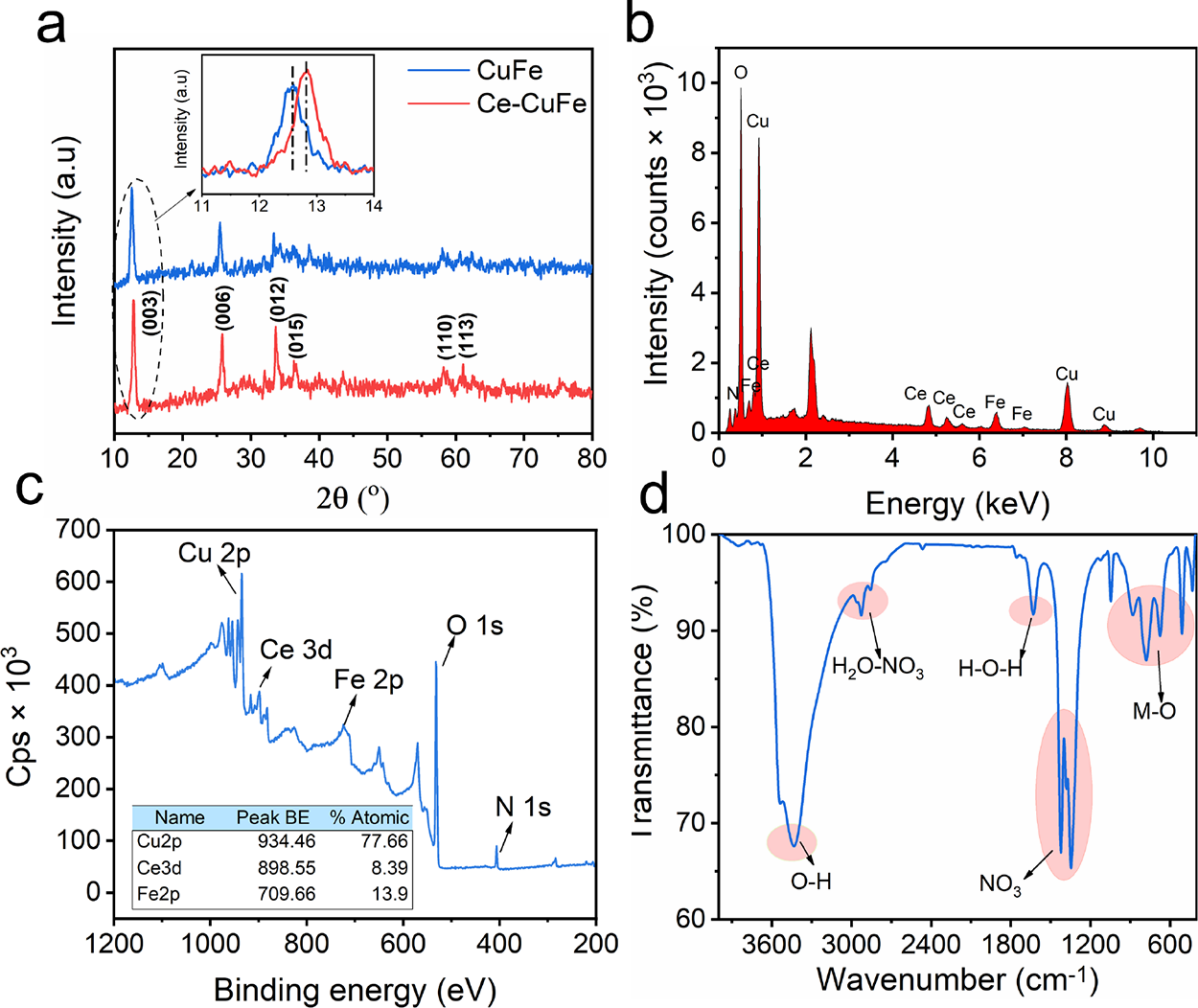
(a) X-ray diffraction (XRD), (b) energy-dispersive spectroscopy, (c) X-ray photoelectron spectroscopy (XPS), and (d) Fourier-transform infrared (FTIR) analyses of the Ce–CuFe catalyst.

The XRD pattern of CuFe (Figure 3a) showed reflection peaks at 12.77, 25.70, 33.68, 35.24, 58.28, and 61.18°, which are assigned to the (003), (006), (012), (015), (110), and (113) crystalline planes, respectively. The observed peaks agree with the characteristic XRD pattern of lamellar structure and stacking planes inherent to the layered double hydroxide configuration.^32^ The XRD pattern of the Ce–CuFe catalyst indicated that Ce was successfully incorporated into the crystalline lattice of the layered catalyst. Furthermore, Ce doping caused a small shift in the (003) peak position, which is consistent with the previous literature.^33−35^ The average crystal size of the Ce–CuFe catalyst was calculated to be 7.57 nm using the Debye–Scherrer formula.^36^

According to the EDS spectrum of the Ce–CuFe catalyst (Figure 3b), the composition includes Ce, Cu, and Fe elements from the hydroxide layer, along with the presence of O and N elements, indicating the interlayer region’s contribution to the catalyst’s elemental makeup. The XPS survey spectrum of the Ce–CuFe catalyst surface confirmed the presence of Cu, Ce, Fe, O, and N elements (Figure 3c). The quantitative determination of the elemental composition revealed that the atomic percentages of Cu, Fe, and Ce in the catalyst were 77.66, 13.9, and 8.39%, respectively. Notably, the molar ratio of Cu:(Fe+Ce) was calculated to be 3.4:1, which closely aligns with the ratios of precursor materials used during the synthesis. These findings from the XPS analysis support the composition of our catalyst, demonstrating the effectiveness of our synthesis approach.

In the FTIR spectra of the Ce–CuFe catalyst (Figure 3d), the wide peak around 3433 cm^–1^ was due to the O–H stretching mode of the hydroxyl groups in the cationic layers and the interlayer water.^20^ The peak at 1629 cm^–1^ is assigned to the bending mode of the water molecules. Bands related to the metal–oxygen vibration modes appeared below 1000 cm^–1^.^37^ The strong peaks between 1530 and 1185 cm^–1^ were caused by the stretching vibration of the interlayer NO_3_ anion of the layered catalyst. The small peaks located between 2988 and 2837 cm^–1^ were attributed to the H_2_O-NO_3_ bridging mode.^20^

### 3.2 Catalytic Activity of Ce–CuFe Catalyst on *o*-Xylene Oxidation

**Figure 4:**
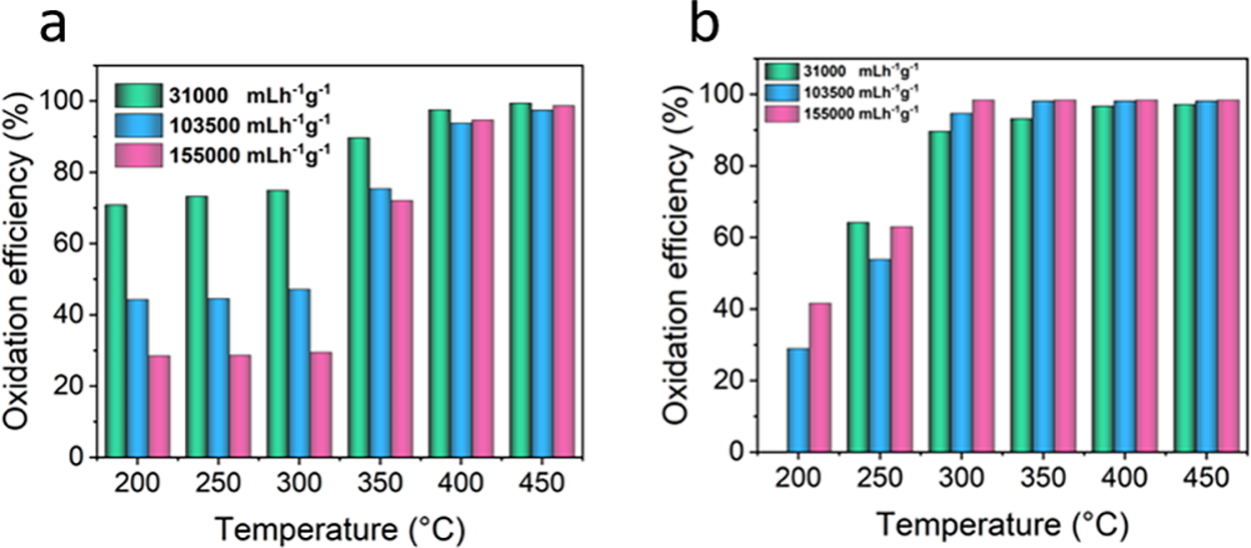
Effect of temperature on the catalytic oxidation of (a) *o*-xylene and (b) *N*,*N*-dimethylacetamide (DMAC) by the Ce–CuFe catalyst at different space velocities.

The catalytic oxidation of *o*-xylene using the Ce–CuFe catalyst is shown in Figure 4. The experiments were conducted at three different space velocities, ranging from 31000 to 155000 mLh^–1^g^–1^, at temperatures between 200 and 450 °C. At all the space velocities, *o*-xylene oxidation followed the same trend: a relatively stable behavior up to 300 °C, followed by a substantial increase at 300–400 °C (Figure 4a). The *o*-xylene oxidation efficiency was almost the same for all flow rates at a maximum temperature of 450 °C, with oxidation efficiencies close to 99%. The effect of the flow rate on catalytic oxidation was more pronounced at lower temperatures (<400 °C), where it is noted that the increase in space velocity decreases the oxidation efficiency of *o*-xylene. For instance, *o*-xylene oxidation efficiencies were measured to be 90, 76, and 73% at 31000, 103500, and 155000 mLh^–1^g^–1^, respectively, at 350 °C. As the space velocity increased, the contact time between the Ce–CuFe catalyst and *o*-xylene decreased, resulting in a lower oxidation efficiency. A lower catalytic *o*-xylene oxidation efficiency was also reported at a higher space velocity using CeO_2_, which is consistent with the results of this study.^38^ In conclusion, the Ce-based catalyst has great potential, providing over 99% *o*-xylene oxidation at an appropriate temperature, regardless of the space velocity.

Examples of catalytic oxidation processes using different catalysts are given in Table 1. However, all of the catalytic oxidation experiments in Table 1 were carried out with lower *o*-xylene concentrations compared to the present work. Only three studies reached around 90% oxidation efficiency. Compared to the studies that had 90% (285 °C)^39^ and 100% (360 °C)^40^ oxidation efficiencies, the present work had 129 and 19 times higher space velocities, respectively. Moreover, the *o*-xylene concentration was 11 times higher compared to the study that had 95% oxidation efficiency at 300 °C.^41^ Therefore, in the present work, it was possible to oxidize *o*-xylene with a higher concentration at higher space velocity.

**Table 1:** Overview of Operation Conditions and Efficiencies of Different Xylene Oxidation Processes

**catalyst**	**xylene (ppm)**	**temp. (°C)**	WHSV* (mLg^**–1**^**h**^**–1**^**)**	**oxidation (%)**	**reference**
V/TiO_2_		360	1631	100	(40)
Pt/SBA-15		365	90000	35	(42)
CeCuCoO	600	300	32000	95	(41)
Au/Co_3_O_4_	85	300	14690	68	(43)
Pt/Co_3_O_4_	150	285	240	90	(39)
Ce-doped CuFe	6536	450	31000	96	present work

Three different mechanisms have been proposed for the oxidation of *o*-xylene using Ce-doped catalysts. One possibility is that *o*-xylene reacted with oxygen molecules that were activated on the catalyst surface and directly oxidized to CO_2_ and H_2_O. Alternatively, *o*-xylene was initially oxidized to *o*-methyl benzaldehyde, which was then adsorbed by the reactive oxygen species on the catalyst surface and further oxidized to CO_2_ and H_2_O.^41^ According to the Mars Van Krevelen degradation mechanism, *o*-xylene is first oxidized to *o*-methyl benzaldehyde, which is then oxidized to methyl benzoic acid and finally converted to CO_2_ and H_2_O.^44,45^ The first step of the Mars Van Krevelen mechanism is the creation of an oxygen vacancy with the reaction of the lattice oxygen on the catalyst surface. In the second step, oxygen from the gas phase is adsorbed onto the catalyst surface and dissociates, which returns the catalyst to its oxidized state. Finally, the Ce oxide phase in the catalyst decomposes and breaks the bonds of the intermediate products to form CO_2_ and H_2_O.^41^ According to GC analysis, no intermediate product was detected in the samples, suggesting direct conversion of *o*-xylene to CO_2_ and H_2_O, which could be a possible scenario for *o*-xylene catalytic oxidation in our study (data not shown).

### 3.3 Catalytic Activity of Ce–CuFe Catalyst on DMAC Oxidation

The catalytic oxidation of DMAC experiments was conducted under the same conditions as those for o-xylene oxidation (Figure 4b). The space velocity showed a limited effect on the oxidation of DMAC in all test temperatures, except for the temperature of 200 °C, at which almost no catalytic oxidation occurred at 31000 mLh^–1^g^–1^. Increasing the space velocity to 103500 and 155000 mLh^–1^g^–1^ resulted in DMAC oxidation efficiencies of 29 and 42%, respectively. In general, a sharp increase was observed when the temperature was increased from 200 to 300 °C, followed by a stable behavior to 450 °C. Implementing the process at 400–450 °C yielded DMAC efficiencies of approximately 99%, regardless of the space velocity. The observed fluctuation in oxidation efficiency at 250 °C could be attributed to several factors. One possible explanation could be the mutual effect of reaction kinetics and mass transfer limitations.^46^ The increase in CO_2_ production within the column during the reaction could potentially result in the blockage of O_2_ due to increased CO_2_ adsorption on the catalyst surface.^47^ Fluctuations in the efficiency of oxidation processes have been reported due to the clogging of active sites on the catalyst surface. This phenomenon occurs when there is a substantial formation of CO_2_ at flow rates that restrict mass transfer and at temperatures exceeding 150 °C.^48^ At a space velocity of 103500 mLh^–1^g^–1^, the increased rate of DMAC transport to the catalyst surface might lead to higher reactant concentrations, causing competitive adsorption or partial surface coverage and consequently reduced catalytic efficiency. However, at 155000 mLh^–1^g^–1^, improved mass transport could facilitate the accessibility of reactants to active sites, leading to increased catalytic activity.

Previous catalytic oxidation studies have shown that mass transfer limitations affect catalytic efficiency.^46^ At high space velocities, external diffusion was found to be negligible.^49^ On the other hand, increasing the space velocity increases the amount of oxygen reaching the catalyst.^50^ Oxygen also provides oxidation at the moment of contact between the solvent and the catalyst surface, where oxygen is held. DMAC has a structure containing less carbon than that of xylene, and the amount of oxygen required for complete oxidation is lower. Although the amount of oxygen delivered at a space velocity of 31000 mLh^–1^g^–1^ was insufficient for DMAC oxidation, a higher oxidation efficiency was obtained by increasing the amount of oxygen delivered along with the increase in the flow rate. In the literature, the catalytic oxidation of DMAC has not been previously investigated. Furthermore, studies have been conducted using liquid DMAC. Among the studies listed in Table 2, the highest removal efficiencies were observed for the hybrid Fenton and catalytic ozonation processes. However, considering the experimental conditions of the present study, which was a gaseous-phase system, direct comparison with these studies is not feasible.

**Table 2:** Overview of Operation Conditions and Efficiencies of Different Processes in *N*,*N*-Dimethylacetamide (DMAC) Degradation

**process**	**catalyst**	**anode/catode**	**DMAC (mg/L)**	**O**_**3**_**(mg/L)**	**H**_**2**_**O**_**2**_**(mg/L)**	**removal (%)**	**reference**
catalytic ozonation	CuFe_2_O_4_		200	4.6		95.4	(23)
ozonation			200	54		27	(51)
Fenton	FeSO_4_·7H_2_O		2000		2000	95.8	(52)
photo-Fenton	FeSO_4_·7H_2_O		250		200	90	(53)
electro-Fenton		iron plates	250		200	96.3	(53)
photo-electro-Fenton		iron plates	500		1500	99	(53)
electrolysis		iron plates	200			2	(51)
electrolysis-ozone		iron plates	200	54		89	(51)

### 3.4 Effect of Ce Doping on the Oxidation of *o*-Xylene and DMAC

**Figure 5:**
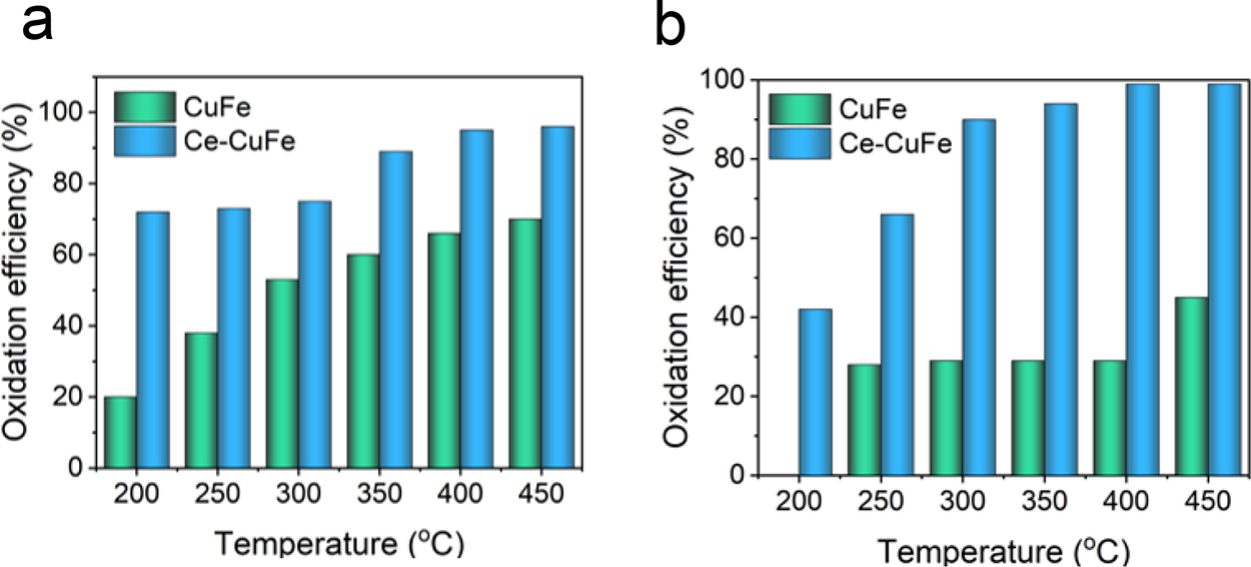
Performance comparison of CuFe and Ce–CuFe catalyst on the catalytic oxidation of (a) *o*-xylene (WHSV = 31000 mLh^–1^g^–1^) and (b) DMAC (WHSV = 155000 mLh^–1^g^–1^) at different temperatures.

To examine the effect of Ce doping on *o*-xylene oxidation, the experiment was performed at 31000 mLh^–1^g^–1^, which was the space velocity with the highest oxidation performance of the Ce–CuFe catalyst (Figure 5a). The presence of Ce in the CuFe catalyst considerably affected the oxidation efficiency of both solvents. In the oxidation of *o*-xylene at 200 °C, the Ce–CuFe catalyst achieved 70% removal efficiency, which decreased to 20% when the undoped CuFe catalyst was used. Across all tested temperatures, the catalytic oxidation performance of the Ce-doped CuFe catalyst was superior to that of the undoped catalyst. To examine the effect of Ce doping on DMAC oxidation, the experiment was performed at a space velocity of 155000 mLh^–1^g^–1^, which was the space velocity with the highest oxidation performance, and results are given in Figure 5b. Similar to the *o*-xylene oxidation, the presence of Ce in the CuFe catalyst positively affected the oxidation performance of DMAC. For instance, the oxidation efficiencies of Ce-doped and undoped CuFe catalysts for DMAC were 42 and 0%, respectively, at 200 °C, respectively. In the DMAC oxidation at 450 °C, the Ce–CuFe catalyst achieved 99% efficiency, which was almost twice the efficiency of undoped CuFe (45%).

The high performance of the Ce–CuFe catalyst in the oxidation of both VOCs is attributed to its high oxygen-holding capacity. Cerium oxide has many oxygen vacancies, a high tendency to store oxygen, and strong interactions with other metals, and its oxidation level can be easily altered. A diesel soot combustion study with Ce-based catalysts showed that Ce ions hold oxygen atoms and store them as surface-oxygen complexes.^54^

### 3.5 Effect of Humidity on the Catalytic Activity of the Ce–CuFe Catalyst

**Figure 6:**
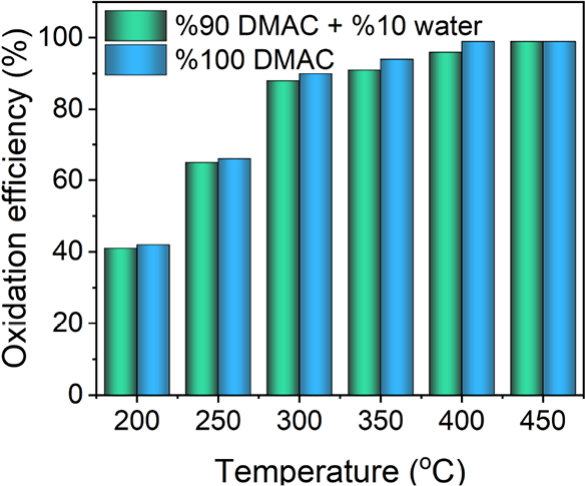
Effect of humidity on the catalytic oxidation of *N*,*N*-dimethylacetamide (DMAC) by Ce–CuFe oxidation at WHSV = 155000 mLh^–1^g^–1^.

The effect of humidity on the catalytic activity of the Ce–CuFe catalyst was tested in a solution containing 10% water and 90% DMAC. The study was performed at a space velocity of 155000 mLh^–1^g^–1^, which was the condition that yielded the highest DMAC oxidation efficiency. As shown in Figure 6, humidity has a limited effect on the catalytic oxidation of DMAC at different temperatures. For instance, the Ce–CuFe catalyst achieved 42 and 39% oxidation efficiencies in DMAC and DMAC/water solutions, respectively, at 200 °C. Regardless of the water content, the oxidation efficiency reached 99% when the temperature was greater than 400 °C.

In studies performed in humid environments, water vapor competes with the active regions of the catalyst, leading to catalyst deactivation.^55^ In a previous study, benzene oxidation efficiency of Co_*x*_NiAlO decreased from 97.2 to 88.8% under humid conditions at 240 °C, whereas no negative effect was observed at 260 °C.^56^ The weakening of the inhibition is attributed to the lower adsorption capacity of water than oxygen onto the catalyst surface at higher temperatures.^57^ The occupation of catalyst active sites by water is a reversible process since these sites can be regenerated upon the removal of water vapor.^58^

### 3.6 Reusability of the Ce–CuFe Catalyst

**Figure 7:**
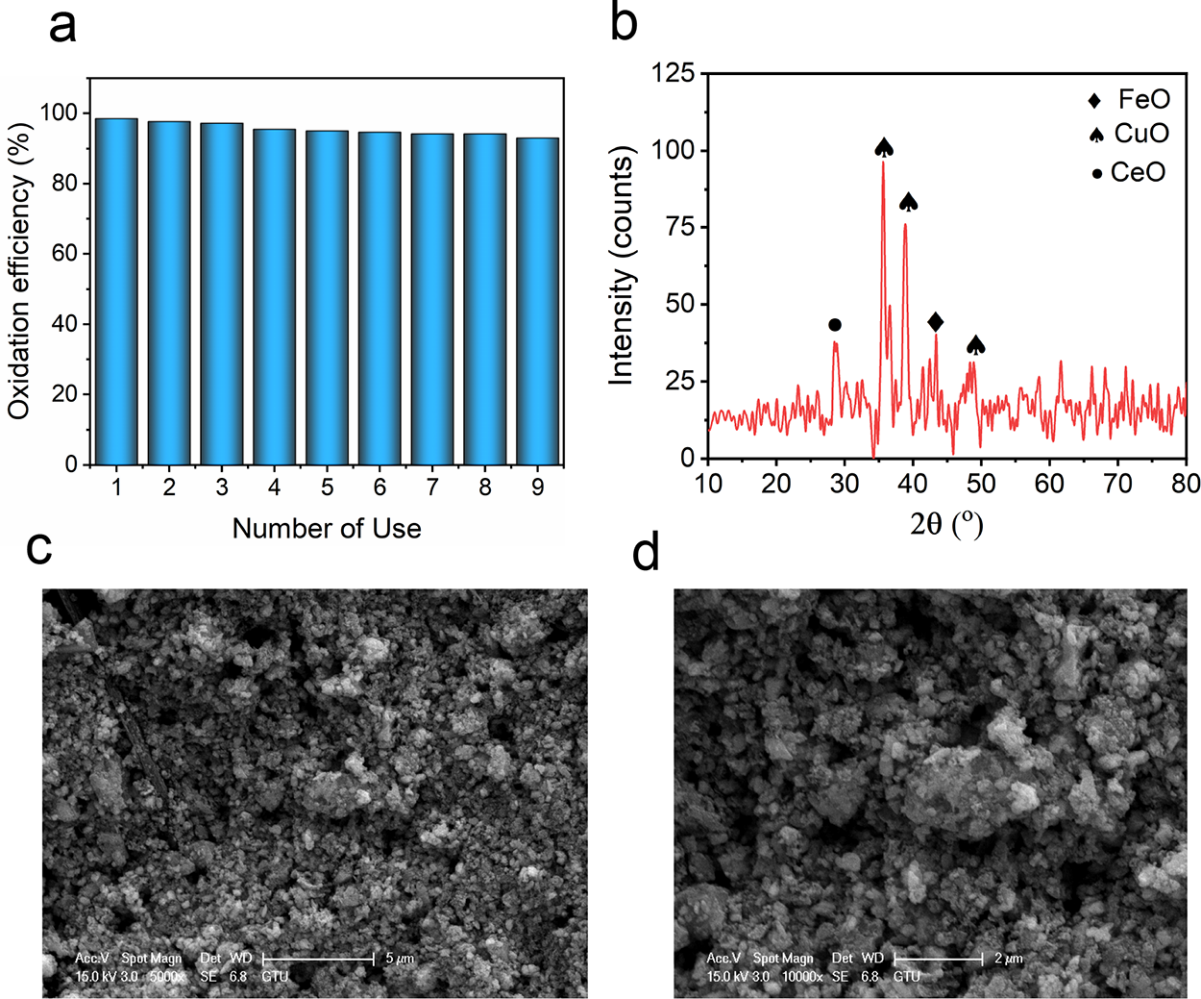
(a) Reusability Ce–CuFe catalyst in the catalytic oxidation of DMAC, (b) XRD profile, and (c, d) SEM images of the Ce–CuFe after the ninth use. Experimental conditions: WHSV = 155000 mLh^–1^g^–1^ and temperature = 300 °C, reaction duration= 6 h).

Figure 7a shows the reusability studies performed at a space velocity of 155000 mLh^–1^g^–1^ at 300 °C for DMAC oxidation. These operating conditions were selected because there was no increase in oxidation performance at temperatures higher than 300 °C, and 155000 mLh^–1^g^–1^ was the highest oxidation efficiency for DMAC. The Ce–CuFe catalyst exhibited outstanding reusability performance, with only a 5.6% efficiency loss after nine reuse cycles. The minor performance loss of the catalyst may be attributed to different factors. When the catalyst was used for repeated runs, the amount of solvent that contacted the catalyst surface increased, which filled the active sites of the catalyst. This resulted in a decrease in the amount of stored oxygen on the catalyst surface and a deterioration in the efficiency of catalytic oxidation.^59^ Another reason could be the mass loss of the catalysts in the temperature range of 190–650 °C because of the gasification of the catalyst’s interlayer anions at temperatures > 200 °C.^60^ Furthermore, ion loss occurs due to water formation.^61^ The water that forms during oxidation is attached in the form of water–carbonate hydroxyl or water-doped ion-hydroxyl. Repeated use of the catalyst increases the amount of water formed and reduces the activity of the catalyst by attaching to the active ions.^61^ The changes in the crystalline structure and morphological properties of the Ce–CuFe catalyst after the ninth reuse were also investigated by XRD and SEM analyses. XRD and SEM analyses of the catalyst after the ninth cycle have provided insightful observations. Notably, the XRD pattern shows alterations in the catalyst’s structure (Figure 7b). The original layered double hydroxide structure of the material appears to have been transformed into a new phase known as a layered double oxide with lower crystallinity. This transformation was expected due to the elevated reaction temperatures, which led to the removal of water molecules present within the interlayers of the layered catalyst. The new peaks observed in the XRD pattern confirm the formation of a new oxide phases. The SEM image of the used catalyst shows aggregated particles that agree with the morphology of layered double oxides (Figure 7c,d).

## 4 Conclusions

Ce–CuFe-layered material was developed as an effective, affordable, and durable catalyst for the oxidation of DMAC and *o*-xylene. According to structural and morphological characterization methods, the material has a two-dimensional flakelike shape, and the layered structure was preserved following Ce doping into the cationic layers. While the increase in the space velocity generally affects DMAC oxidation positively, *o*-xylene oxidation was negatively affected. At the lowest temperature (200 °C), the undoped CuFe catalyst could only oxidize 20% of the *o*-xylene, while zero oxidation was achieved for the DMAC. On the other hand, the Ce–CuFe catalyst achieved 70 and 72% oxidation efficiencies for *o*-xylene and DMAC, respectively, at 200 °C. The Ce–CuFe catalyst was found capable of removing DMAC and *o*-xylene with an efficiency of more than 95% at 450 °C. However, the oxidation efficiencies of *o*-xylene and DMAC were 70 and 45%, respectively, for an undoped CuFe catalyst at 450 °C. The activity of the Ce–CuFe catalyst in the oxidation of DMAC remained unaffected by the presence of humidity in the temperature range of 200–400 °C. The performance loss of the Ce–CuFe catalyst was only 5.6% after repetitive oxidation tests. All of these positive features might make Ce-doped CuFe preferable for industrial catalytic oxidation applications.
